# Crusted Scabies, a Neglected Tropical Disease: Case Series and Literature Review

**DOI:** 10.3390/idr14030051

**Published:** 2022-06-16

**Authors:** Nurdjannah Jane Niode, Aryani Adji, Shienty Gazpers, Renate Tamara Kandou, Herry Pandaleke, Dwi Martina Trisnowati, Christy Tumbelaka, Elrovita Donata, Fauziyyah Nurani Djaafara, Hendrix Indra Kusuma, Ali A. Rabaan, Mohammed Garout, Souad A. Almuthree, Hatem M. Alhani, Mohammed Aljeldah, Hawra Albayat, Mohammed Alsaeed, Wadha A. Alfouzan, Firzan Nainu, Kuldeep Dhama, Harapan Harapan, Trina Ekawati Tallei

**Affiliations:** 1 Department of Dermatology and Venereology, Faculty of Medicine, Kandou General Hospital, Universitas Sam Ratulangi, Manado 95115, Indonesia; niodejane@unsrat.ac.id (N.J.N.); aryaniadji@gmail.com (A.A.); shienty@yahoo.com (S.G.); renate.kandou@yahoo.com (R.T.K.); herrypandaleke53@gmail.com (H.P.); martinawang1803@gmail.com (D.M.T.); cbtumbelaka@gmail.com (C.T.); elrovita.donata@hotmail.com (E.D.); 2 North Sulawesi General Hospital, Manado 95115, Indonesia; fauziyyahdj@gmail.com; 3 Medical Research Unit, School of Medicine, Universitas Syiah Kuala, Banda Aceh 23111, Indonesia; hendrixkusuma.hk@gmail.com; 4 Faculty of Mathematics and Natural Sciences, Universitas Syiah Kuala, Banda Aceh 23111, Indonesia; 5 Faculty of Tarbiyah and Teacher Training, Universitas Islam Negeri Ar-Raniry, Banda Aceh 23111, Indonesia; 6 Molecular Diagnostic Laboratory, Johns Hopkins Aramco Healthcare, Dhahran 31311, Saudi Arabia; arabaan@gmail.com; 7 College of Medicine, Alfaisal University, Riyadh 11533, Saudi Arabia; 8 Department of Public Health and Nutrition, The University of Haripur, Haripur 22610, Pakistan; 9 Department of Community Medicine and Health Care for Pilgrims, Faculty of Medicine, Umm Al-Qura University, Makkah 21955, Saudi Arabia; magarout@uqu.edu.sa; 10 Department of Infectious Disease, King Abdullah Medical City, Makkah 43442, Saudi Arabia; dr_mas82@yahoo.com; 11 Department of Pediatric Infectious Disease, Maternity and Children Hospital, Dammam 31176, Saudi Arabia; hanihm@gmail.com; 12 Department of Infection Control, Maternity and Children Hospital, Dammam 31176, Saudi Arabia; 13 Preventive Medicine and Infection Prevention and Control Department, Directorate of Ministry of Health, Dammam 32245, Saudi Arabia; 14 Department of Clinical Laboratory Sciences, College of Applied Medical Sciences, University of Hafr A Batin, Hafr A Batin 39524, Saudi Arabia; mmaljeldah@uhb.edu.sa; 15 Department of Infectious Disease, King Saud Medical City, Riyadh 7790, Saudi Arabia; hhalbayat@gmail.com; 16 Division of Infectious Disease, Department of Medicine, Prince Sultan Military Medical City, Riyadh 11159, Saudi Arabia; mohalsaeed@live.com; 17 Department of Microbiology, Faculty of Medicine, Kuwait University, Safat 13110, Kuwait; alfouzan.w@hsc.edu.kw; 18 Microbiology Unit, Department of Laboratories, Farwania Hospital, Farwania 85000, Kuwait; 19 Department of Pharmacy, Faculty of Pharmacy, Hasanuddin University, Makassar 90245, Indonesia; firzannainu@unhas.ac.id; 20 Division of Pathology, ICAR-Indian Veterinary Research Institute, Bareilly 243122, India; kdhama@rediffmail.com; 21 Tropical Disease Centre, School of Medicine, Universitas Syiah Kuala, Banda Aceh 23111, Indonesia; 22 Department of Microbiology, School of Medicine, Universitas Syiah Kuala, Banda Aceh 23111, Indonesia; 23 Department of Biology, Faculty of Mathematics and Natural Sciences, Universitas Sam Ratulangi, Manado 95115, Indonesia

**Keywords:** crusted scabies, case reports, literature review, mortality, outbreak

## Abstract

Crusted scabies is a rare form of scabies that presents with more severe symptoms than those of classic scabies. It is characterized by large crusted lesions, extensive scales, thick hyperkeratosis, and contains a large number of highly contagious itch mites. Crusted scabies is more prevalent in immunocompromised, malnourished, and disabled individuals. This disease has been linked to a variety of health problems, including delayed diagnosis, infection risk, and high mortality, mainly from sepsis, and it has the potential to cause an outbreak due to its hyper-infestation, which makes it highly infectious. This article reports three cases of crusted scabies in North Sulawesi, Indonesia. Recent updates and a comprehensive review of the literature on the disease are also included, emphasizing the critical importance of early diagnosis and effective medical management of patients, which are necessary to prevent the complications and spread in communities.

## 1. Introduction

Scabies is a skin disease that has been identified as a neglected tropical disease (NTD) [1]. This is caused by the infection of the human-specific ectoparasite *Sarcoptes scabiei* var. *hominis* and manifests as intolerably intense itching with various severities of skin lesions [2]. Scabies has spread globally, mainly in tropical, populous, and impoverished regions, as well as in areas with limited healthcare resources [3]. Crusted scabies (previously known as “Norwegian” scabies) is a rare form of scabies that presents with more severe symptoms than classic scabies. It is characterized by large crusted lesions, generalized scales, thick hyperkeratosis, and contains an abundance of highly contagious mites that can be life-threatening [4]. The World Health Organization (WHO) estimates there are 200 million scabies cases at any time globally and Indonesia has the greatest scabies burden among 195 countries [1,5]. There is no prevalence data for crusted scabies, either globally or in Indonesia.

Crusted scabies is most frequently reported in immunocompromised, malnourished, and disabled individuals, as well as in patients with systemic or potent topical glucocorticoids, organ transplant recipients, human immunodeficiency virus (HIV)-infected or human T-lymphotropic virus-1 (HTLV-1)-positive individuals, and patients with hematologic malignancies [6,7]. However, a report also identified the disease in immunocompetent indigenous Australians [8]. Crusted scabies is a significant public health problem, affecting a broader audience, including families and communities [9], and spreading continuously through direct skin-to-skin contact [4]. Due to delayed diagnosis, crusted scabies could cause multiple complications such as secondary impetigo, cellulitis, and sepsis with a high mortality rate [10]. Owing to its hyper-infestation, it has the potential to cause an outbreak, making it extremely contagious [11,12]. In addition to direct contact with carriers, crusted scabies can be transmitted via direct or indirect contact with contaminated materials such as bedding and clothing [13]. To prevent the spread of the disease in communities, early and accurate diagnosis, effective medical management of patients and healthcare settings, and specific principles and strategies for disease management are required.

In the present study, three cases of crusted scabies in North Sulawesi, Indonesia, were presented. The most recent updates and a comprehensive literature review are also included, emphasizing the critical importance of early diagnosis and effective medical management of patients, which are critical for preventing disease complications and containing the spread of the disease in communities.

## 2. Materials and Methods

Three crusted scabies cases were reported: (1) a male, 79 years old; (2) a male, 45 years old; and (3) a female, 62 years old. All patients were confirmed to have comorbid conditions. Written informed consent for publication was obtained from all the families.

A literature review was also conducted across various databases, including the National Library of Medicine (PubMed), Google Scholar, Elsevier, the Public Library of Science (PLoS), and Semantic Scholar. Literature searches were set up using the keyword “crusted scabies” filtered only for publications within the last ten years (2011–2021). Only articles published in English were included, varying between case reports and retrospective/prospective case series. The full texts of potentially eligible studies were obtained and assessed for eligibility by the authors. Articles with incomplete data were excluded from the analysis.

## 3. Results

### 3.1. Case Reports

**Figure 1:**
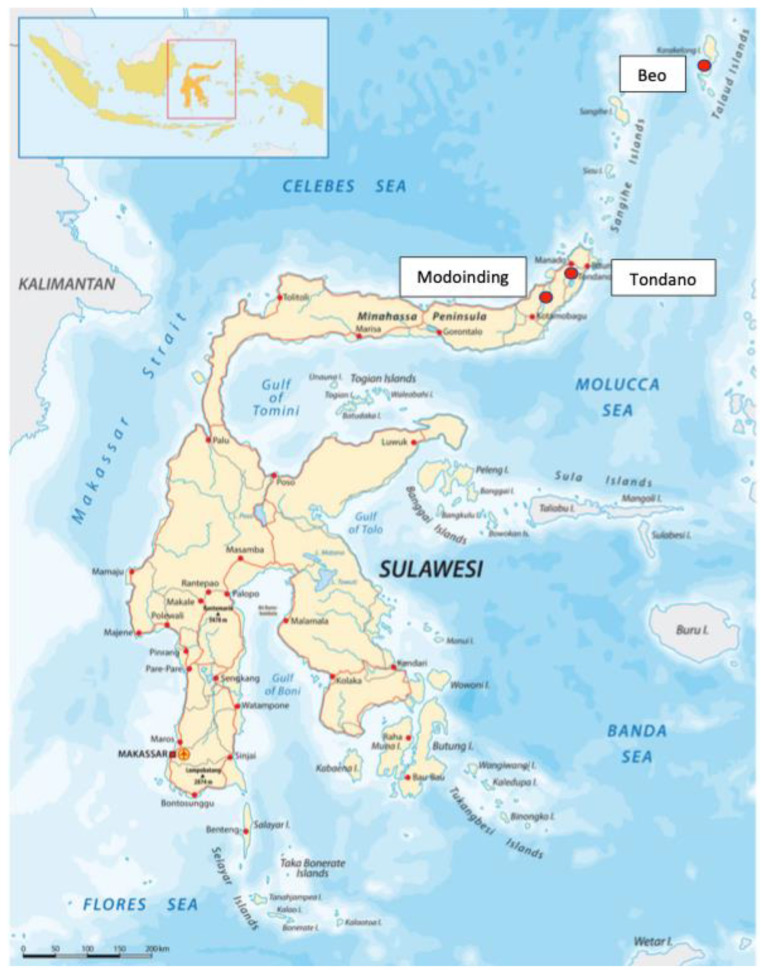
Map of Sulawesi indicating the source of cases in North Sulawesi.

Three cases of crusted scabies in this study occurred in the span of six months within the same year in three different places in North Sulawesi Province. The patients were from Beo, Modoinding, and Tondano (Figure 1).

#### 3.1.1. Case 1

**Figure 2:**
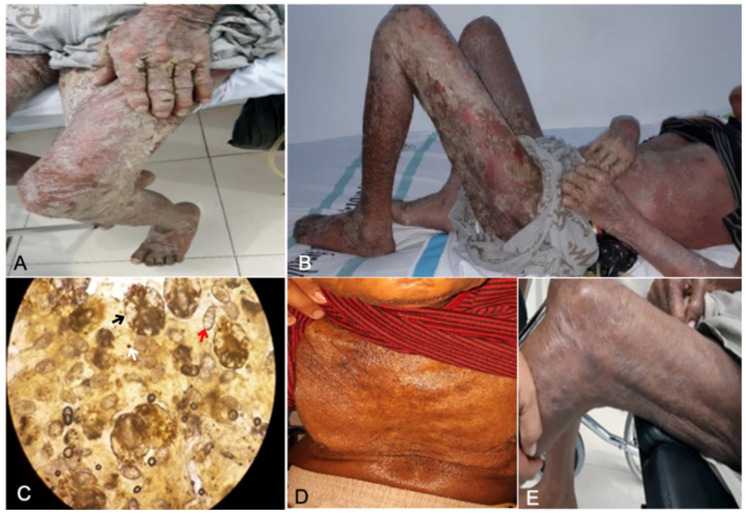
Patient of Case 1 showing diffuse erythematous maculopapular, generalized gray-yellow crusts (**A**,**B**); mineral oil scraping showing *Sarcoptes* *scabiei* mite (black arrow), eggs (red arrow), and feces (white arrow) (**C**); and clinical recovery after four weeks of therapy (**D**,**E**).

A 79-year-old male from Beo Village, Talaud Island, North Sulawesi presented with thick gray-yellow crusts on the skin throughout the body and itching at night. The disease had manifested for a month. Family members living under the same roof also had similar symptoms. The patient had a history of stroke and muscle weakness on the right side of the body. Physical examination revealed generalized erythematous maculopapular rashes covered by grayish-white-yellow crusts (Figure 2A,B). Microscopic examination at 100× magnification revealed numerous mites, eggs, and scybala in the skin sample from between the fingers (Figure 2C). Topical therapies of permethrin 5% and a keratolytic agent (salicylic acid 3%) were administered. Permethrin cream was applied throughout the body every night (12 h duration) for a week and then continued two times a week for three weeks (i.e., four weeks in total). Salicylic acid 3% in white petrolatum ointment was applied to areas with crusty lesions, such as the hands and thighs, every day for 13 days. Potentially contagious family members were also treated with permethrin once a week and continued until contact with infected patients was terminated, or patients recovered fully.

The measures to control disease transmission were delivered, including education on the disease to patients and families, appropriate treatments for potentially infected individuals, and the elimination of mites, including cleaning contaminated clothing, home appliances, and other fomites. On the final examination, no previous symptoms of crusted scabies, as well as mites, eggs, or scybala, were found in the patient. Complete regression was confirmed after one month of treatment (Figure 2D,E).

#### 3.1.2. Case 2

**Figure 3:**
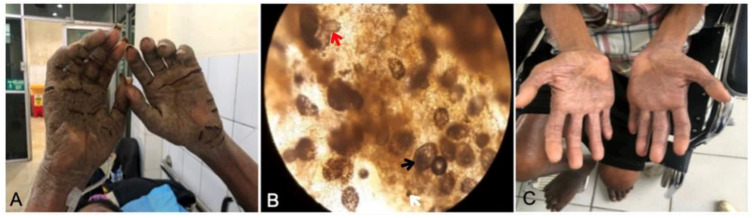
Patient of Case 2 showing gray-brown, slightly yellowish crusts on the both palmar regions (**A**); mineral oil scraping showing *Sarcoptes* *scabiei* mite (black arrow), eggs (red arrow), and feces (white arrow) (**B**); and clinical recovery after three weeks of therapy (**C**).

A 45-year-old man from the Tondano region, Minahasa Regency, North Sulawesi, presented with thick gray-brown, slightly yellowish crusts on the skin throughout the body, in particular on the palm (without itch). The disease had manifested for five months. Family members living under the same roof also had itching at night. The patient had lumbar trauma and paraplegia previously and had uncontrolled diabetes. Physical examination revealed grayish-white-yellow crusts, primarily on both palmar regions (Figure 3A). Microscopic examination at 100× magnification with mineral oil revealed multiple mites, eggs, and scybala in skin samples from between the fingers (Figure 3B). Topical therapies of permethrin 5% and keratolytic (salicylic acid 3%) were administered. Permethrin cream was applied throughout the body every night (12 h duration) for a week and then continued two times a week for three weeks. The patient was administered permethrin for four weeks. Salicylic acid 3% in the white petrolatum ointment was applied to areas with crusty lesions, such as the hands and thighs, every morning for 13 days. Potentially contagious family members were treated with permethrin once a week and continued until contact with infected patients was terminated, or patients had recovered fully. Measures to control disease transmission were delivered, including education on the disease to patients and families, appropriate treatments for potentially infected individuals, and elimination of mites, including cleaning contaminated clothing, home appliances, and other fomites.

On the final examination at the end of the treatment, no previous symptoms of crusted scabies were found on the patient’s skin, and the components of mites were absent. Complete regression was confirmed after three weeks of treatment (Figure 3C).

#### 3.1.3. Case 3

**Figure 4:**
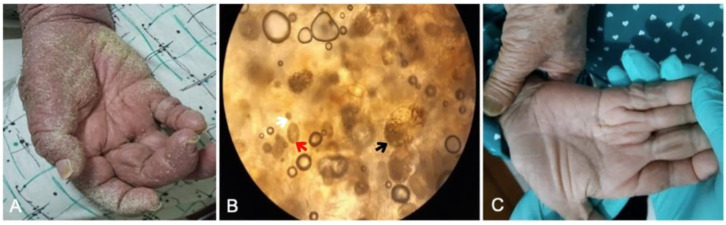
Patient of Case 3 showing gray-yellow crusts on the regio palmar dexter (**A**); Mineral oil scraping showing *Sarcoptes scabiei* mite (black arrow), eggs (red arrow), and feces (white arrow) at 10-times magnification (**B**) and clinical recovery after two weeks of therapy (**C**).

A 62-year-old female from Modoinding Village, West Minahasa Regency, North Sulawesi Province, presented with thick, yellow, slightly gray crusts on the skin throughout the body, especially on the palm (without itch). The disease had manifested for two months. Family members living with the patient also had itching at night. The patient had a history of stroke and muscle weakness on the right side of the body. On physical examination, slightly yellow-gray crusts primarily on the right palmar region were found (Figure 4A). Microscopic examination of the skin sample from between the fingers at 100× magnification showed multiple mites, eggs, and feces (Figure 4B). The patient was treated with topical permethrin 5% throughout the body every night (12 h duration) for a week, followed by twice in the following week. The subject was administered permethrin for two weeks. The family members were also treated with permethrin once a week and continued until contact with infected patients was terminated, or the patient had fully recovered. Measures to control disease transmission were delivered, including education on the disease to patients and families, appropriate treatments for potentially infected individuals, and elimination of mites, including cleaning contaminated clothing, home appliances, and other fomites.

On the final examination, no previous symptoms of crusted scabies were found in the patient’s skin, and components of mites on the skin were absent. Complete regression was confirmed after two weeks of treatment (Figure 4C).

The summary of the reported crusted scabies cases from North Sulawesi is presented in Table 1.

### 3.2. Literature Review

A total of 59 eligible peer-reviewed articles on crusted scabies published in the last ten years were assessed in this study. There were 22 articles which contained case reports, and one contained a prospective case series of crusted scabies. The summary of crusted scabies cases from the literature together with our three cases is presented in Table 2. Data from case report studies indicated that 43% of the cases were reported in adults and 34.8% in the elderly with a male:female ratio of 1.1:1. Comorbidities were reported in 69.6% of cases, most of them were diabetes, followed by disorders with defective T cell immune responses such as HIV infection and Down syndrome. Most authors (87%) reported successful treatment of patients using oral ivermectin with or without a topical permethrin combination (20 out of 23 cases). Only a small number (13%) used topical permethrin treatment (3 out of 23 cases).

A prospective case series study [12] found that the median age at risk was 47 years, women were more prevalent than men (59% vs. 41%), and the majority of patients (82%) had comorbid conditions. All of the cases were treated with oral ivermectin and topical scabicide. Overall, most patients achieved complete regression, although some cases resulted in death.

## 4. Discussion

In this present study, all three reported cases of crusted scabies in North Sulawesi were in elderly patients, and all patients had co-occurring medical conditions. They experienced complete regression after treatment with topical permethrin 5% combined with keratolytic agents. Clinical manifestations and treatment outcomes were not significantly different from similar cases in the literature. However, the types of treatments administered to patients vary considerably. While the cases in our study were treated with topical therapy (permethrin 5% and keratolytic agents), most of the literature indicates that the disease is best treated with a combination of oral ivermectin and topical therapy.

The earliest report on crusted scabies was published in 1848 by Danielsen and Boeck. Because the disease was discovered in a patient with leprosy in Norway, it was previously referred to as “Norwegian scabies” [14]. Crusted scabies is a severe form of common scabies that is particularly susceptible to hyper-infection by individuals who lack the ability to control scabies mite proliferation. The number of mites is abundant, millions or more, causing extensive skin hyperkeratosis and crusting [7].

Regarding the etiology, the causative agent of crusted scabies and classic scabies is the same, *S. scabiei* var. *hominis*, belonging to the phylum *Arthropoda*, class *Arachnida*, order *Ackarina*, and superfamily *Sarcoptes* [36]. This species is an obligate parasite that burrows in the lower stratum corneum of the skin [37]. The life cycle of *S. scabiei* is completed in four stages: egg, larva, nymph (protonymph and tritonymph), and adult [38]. The duration of one life cycle of human itch mites varies, approximately 9–15 days, which may be related to the difficulty in observing mites on the skin in vivo [39]. 

Female adult scabies mites contribute significantly to the pathogenesis of the disease. Mating occurs only once in a lifetime, and males typically die shortly after mating, whereas females live significantly longer. The female mite burrows beneath the skin and spends 1–2 months laying the eggs [40]. Approximately 2–3 days after the eggs are laid, the larvae emerge and cut through the burrows to reach the skin surface to mate and reproduce [41]. Female mites construct their characteristic serpentine burrows using proteolytic enzymes to soften them and enable them to dig deeper into the stratum corneum of the skin epidermis. Scabies mites can survive in nature for approximately 24–36 h at normal temperature (21 °C and relative humidity of 40–80%), during which time they remain capable of infestation [4].

Suppression of the immune system allows the *Sarcoptes* mites to spread quickly throughout the body, resulting in widespread infestation [42]. Nonetheless, the immunological mechanisms underlying the disease onset are not fully understood [43]. Crusted scabies is more prevalent in individuals with a deficient T cell immune response, decreased cutaneous sensation due to neurological disorders, and decreased ability to remove mites mechanically [34,44]. The same conditions were observed in all three patients in our study. In addition, they had comorbid conditions associated with neurological and cardiovascular disorders, implying that they had physical limitations or a diminished ability to scratch or debride mites directly.

CD8^+^ T cells are crucial skin-infiltrating T cells, and peripheral blood mononuclear cells obtained from crusted scabies patients exhibit a non-protective T helper 2 (TH2) response characterized by increased production of interleukin (IL)-4, IL-5, and IL-13, as well as lower levels of interferon-c (IFNc) [7,43], and a decreased T helper 1 (TH1) cytokine-interferon in response to the active cysteine protease [45]. Levels of total IgA, IgG4, antigen-specific IgE, and eosinophils are also higher in crusted scabies patients [43,45].

Hyperkeratotic skin lesions are thought to be associated with elevated IL-4 levels in patients with crusted scabies [14]. An imbalance in skin-homing cytotoxic T cells can intensify the dermis of crusted scabies lesional skin by affecting the inflammatory response [7]. When combined with a low B cell count, this condition can result in weakened immune systems incapable of providing effective responses to parasite development [6,34]. Additionally, it is believed that there are genetic predispositions associated with crusted scabies susceptibility that contribute indirectly to its development [44].

Large crusty lesions and thick yellow to brown, creamy-gray skin scales are typical clinical manifestations of crusted scabies [46]. This hyperkeratotic lesion is caused by a high mite concentration, which stimulates excessive keratin production in the stratum corneum [47]. The lesion commonly forms on the hands, feet, neck, scalp, face, trunk, and limbs [48]. Our patients exhibited erythematous plaques that developed into thick brown-yellow-and-gray crusts, especially on the palms, soles, and extensor surfaces. In common and neglected manifestations, such as those seen in our patients, the diagnosis can be made through the symptom of yellow-brown crusts with a “built-up sand” appearance that affect fingers and hands. These thick crusts have the appearance of a “rocky surface,” with deep fissures dividing them into segments against an erythematous background [18].

In terms of diagnosis, crusted scabies should be differentiated from psoriasis, seborrheic dermatitis, atopic dermatitis, hyperkeratotic eczema, dyshidrotic eczema, psoriasis, palmoplantar keratoderma, erythrodermic mycoses fungoides, and Sézary syndrome [44]. The integration of a crusted scabies diagnosis can be supported by a rapid bedside test involving microscopic visualization of mites, eggs, or feces from the skin scrapings of concerned individuals [18]. Dermatoscopy and reflectance confocal microscopy can be used to visualize scabies mites, which have the appearance of a “delta-wing jet” [15,49]. A study compared low-cost videomicroscopes (VMs) and high-resolution videodermatoscope (VD) to diagnose scabies and found that VMs could be used for a definitive scabies diagnosis [50]. In addition, VMs could be used during mass screening, post-therapeutic follow-up and therefore could reduce the cost burden, in particular in low socioeconomic settings [50]. In addition, the typical visualization of scabies mites through histopathological examination consists of serpentine burrows containing female mites in the subcorneal layer of the epidermis. The burrow contains all developmental stages of the mite, including eggs, larvae, nymphs, and adults [14].

Crusted scabies can lead to complications due to secondary bacterial infections. *Staphylococcus aureus* could colonize the burrow and trigger erythroderma and septicemia, and superinfection with *Streptococcus pyogenes*, which causes glomerulonephritis and rheumatic fever [51,52,53]. Crusted scabies has a high mortality rate of 50% in the last five years due to sepsis or secondary infections [54].

Medical management of crusted scabies is challenging for a variety of reasons, including immunocompromised status [20], polymorphous skin lesions [55], an abundance of mites [4], hyperkeratotic lesions [24], and ineffective penetration of topical agents due to thick crusts [56]. Therapy failure is a common [57]. The fundamentals of crusted scabies management include isolation of patients until complete regression [58]; determining an accurate and adequate therapy that includes topical scabicidal, antiectoparasit, and keratolytic agents [14,59]; symptomatic therapy such as antihistamines and antibiotics for secondary infection [14,60]; comprehensive education and accurate information to the patient and the family members [14,60]; and appropriate skin scraping in the first and fourth week after treatment [14].

Information on controlled therapeutic studies for crusted scabies is not yet available, and the best treatment for this disease remains unclear [61]. Nonetheless, several therapeutic regimens can be integrated into a treatment program, such as combination treatment with topical scabicide and oral ivermectin [4,62]. The treatment consisted of 5% topical permethrin cream (recurrent generalized application for seven days, then two times/week until complete regression is acquired) [63] or 25% topical benzyl benzoate combined with oral ivermectin 200 µg per body weight (kg) taken on days 1, 2, 8, 9, and 15 [62].

Topical permethrin is a scabicidal which is a synthetic pyrethrin formulation in a 5% cream, and it works by disrupting the neuronal sodium channel repolarization, causing paralysis and death of the mites [14]. Permethrin is safe for pregnant and lactating women, and can be used in infants two months of age or older [58]. There is no data on permethrin resistance in humans. However, the resistance in animals has been reported [64]. Ivermectin, a semisynthetic anti-helminthic agent derived from *Streptomyces avirmitilis*, is an effective oral scabicidal that inhibits the gamma amino benzoic acid (GABA) [14]. This drug should not be used in pregnant women and in children weighing less than 15 kg [58]. Ivermectin resistance has been reported in human scabies due to some potential mechanisms, including the presence of voltage-gated sodium or ligand-gated chloride channels, glutathione S-transferase and ATP-binding cassette transporters [64]. Keratolytic agents such as 5–10% salicylic acid and 40% urea are needed to remove crusts and are therefore important to reduce the number of mites, and also increase the effectiveness of a topical scabicidal [14].

In severe cases, additional treatment with ivermectin can be added to the routine 22 and 29 days after treatment began [58,65]. In Australia’s tropical Northern Territory, the severity of crusted scabies is graded into scales based on the clinical assessment of four domains: the distribution and extent of crusting; the intensity of skin crusting or shedding; the state of skin condition, including the degree of cracking and pyoderma; and the frequency of past episodes. The scale serves a variety of purposes. Initially, it was developed for hospitalized patients. It is now used to assess and manage crusted scabies disease in a shorter duration than treatment without the grading scale while maintaining equivalent outcomes [66]. In addition to the combination therapy of a topical scabicide and oral ivermectin, another alternative treatment that promises desirable outcomes is using two or more topical agents cyclically or recurrently for three to four cycles [14]. However, there are still many countries that lack ivermectin. Therefore, in our cases, oral ivermectin was substituted with topical scabicide therapy (permethrin 5% and keratolytic agents) for a week continuously and then proceeded with two times/week for three weeks. The results indicated complete regression in all three patients. Besides patients, family members and a person with close interaction with the patient, symptomatic or asymptomatic, also need to be treated simultaneously because multiple cases indicate asymptomatic carriers among individuals who live under the same roof as the concerned patient [4]. Environmental decontamination also plays a vital role in crusted scabies treatment [14]. Clothing, towels, beds, bedsheets, and sofas were cleaned with water at >500 °C or dry-heated at 600 °C for 10 min. Another alternative is to store the contaminated fomites inside plastic bags for 72 h. Floors and furniture can be cleaned with a vacuum cleaner, and non-washable fomites can be cleaned using insecticides [14,67].

There is a new potential management option to prevent the development of scabies, especially in endemic areas and with crusted scabies, and an anti-mite vaccine has been tested in an animal model (rabbits and mites) [68]. However, further studies are warranted before these could be implemented in clinical practices.

## 5. Conclusions

Crusted scabies is a rare variant of classic scabies, characterized by a massive mite infestation. The primary complications caused by crusted scabies are delayed diagnosis, ineffective treatment, secondary infections associated with mortality, recurrent infection, and the source of an outbreak due to its hyper-infestation, making it highly infectious. Therefore, effective and precise diagnosis and medical management are required, encompassing both patients and healthcare settings. Additionally, there is a need for prevention before developing scabies or crusted scabies, such as an anti-mite vaccine, although additional research to develop an effective vaccine is still required.

## Figures and Tables

**Table 1:** Summary of findings from crusted scabies patients in North Sulawesi.

Location	Age (Year)/Sex	Comorbid	Treatment	Outcome
Beo, Talaud Island	79, male	Neurological disorder (stroke)	Topical permethrin 5% cream every day for seven days, continued twice a week for three weeks and keratolytic agents	Complete regression
Tondano, Minahasa	45, male	Metabolic disorder (diabetes mellitus)	Topical permethrin 5% cream every day for seven days, continued twice a week for three weeks and keratolytic agents	Complete regression
Modoinding, West Minahasa	62, female	Neurological disorder (stroke)	Topical permethrin 5% cream every day for seven days, continued twice a week for one week	Complete regression

**Table 2:** Summarized finding from the literature review of crusted scabies.

Country	Age (Year)/Sex	Associated Conditions	Treatment	Outcome	Reference
India	54/male	Diabetes	Oral ivermectin, topical permethrin 5% cream	Complete regression	[15]
Italy	0.3/male	SARS-CoV-2 infection	Oral ivermectin, topical permethrin 5% and urea 5% cream	Complete regression	[16]
India	71/female	None	Oral ivermectin, permethrin cream	Complete regression	[17]
Mexico	38/female	Down syndrome, dehydration	Intravenous fluids, oral ivermectin, topical permethrin 5% cream	Died	[18]
USA	Elderly/female	None	Oral ivermectin, topical permethrin 5% cream	Died (chronic aspiration)	[19]
Ghana	44/female	HIV	Oral ivermectin, petroleum jelly, oral amoxycillin clavulanate (concomitant skin infection)	Complete regression	[20]
Canada	65/male	Renal transplant recipient	Oral ivermectin	Complete regression	[21]
USA	56/male	None	Oral ivermectin and topical permethrin, debridement	Complete regression	[22]
USA	11/female	Down syndrome	Oral ivermectin, topical permethrin 5% cream, oral clindamycin (concomitant skin infection)	Improvement	[23]
Canada	0.9/male	None	Topical permethrin 5% cream	Complete regression	[24]
Italy	63/male	Bone marrow tranplantation	Oral ivermectin, benzyl benzoate ointment	Complete regression	[25]
USA	53/female	None	Oral ivermectin, topical permethrin 5% cream	Complete regression	[13]
Portugal	87/female	Mild vascular dementia	Oral ivermectin, topical sulfur 6% ointment, keratolytic creams	Complete regression	[26]
USA	34/male	HIV	Oral ivermectin, topical permethrin 5% cream, oral trimethoprim-sulfamethoxazole, and oral combination elvitegravir-cobicistat-emtricitabine-tenofovir (for HIV)	Complete regression	[27]
Brazil	19/male	None	Oral ivermectin, deltamethrin solution, oral amoxicillin clavulanate and clindamycin (concomitant skin infection)	Complete regression	[28]
USA	46/female	Down syndrome	Oral ivermectin, topical permethrin 5% cream, oral doxycycline and sulfamethoxazole-trimethoprim (concomitant skin infection)	Complete regression	[29]
Italy	67/male	HIV	Oral ivermectin, topical permethrin 5% cream, emollients, anti-keratolytic	Complete regression	[30]
Brazil	0.3/male	None	Topical permethrin 1% lotion, intravenous antibiotic (concomitant skin infection)	Died	[31]
USA	60/male	Bipolar disorder, schizoaffective disorder, hypertension, hepatitis C, and peripheral neuropathy secondary to alcoholism	Oral ivermectin and topical permethrin cream, oral Bactrim, in addition to the IV cefazolin (concomitant skin infection), debridement	Complete regression	[32]
USA	66/male	Diabetes	Topical permethrin 5% cream	Complete regression	[33]
Japan	90/male	Diabetes	Oral ivermectin, 1% gamma benzene hexachloride (γ-BHC) ointment, crotamiton ointment containing benzyl benzoate	Complete regression	[34]
Mexico	28/female	HIV	Oral ivermectin, topical ointment containing balsam of Peru, precipitate sulfur and benzoate butter	Complete regression	[35]
Darwin, Northern Territory	47 (median age)/female (59%)	Diabetic, kidney failure (dialysis), chronic lung disease, chronic liver disease, HTLV-1, other immunosuppression, no comorbidities	Oral ivermectin together with daily alternating topical scabicides and topical keratolytic cream.	Complete regression (84%), died (16%)	[12]

## Data Availability

No data are associated with this article.
